# Expression of concern: Recyclable mesalamine-functionalized magnetic nanoparticles (mesalamine/GPTMS@SiO_2_@Fe_3_O_4_) for tandem Knoevenagel–Michael cyclocondensation: grinding technique for the synthesis of biologically active 2-amino-4*H*-benzo[*b*]pyran derivatives

**DOI:** 10.1039/d5ra90023a

**Published:** 2025-03-10

**Authors:** Mahdiyeh Partovi, Sobhan Rezayati, Ali Ramazani, Yavar Ahmadi, Hooman Taherkhani

**Affiliations:** a Department of Chemistry, Faculty of Science, University of Zanjan Zanjan 45371-38791 Iran aliramazani@znu.ac.ir aliramazani@gmail.com; b Department of Biotechnology, Research Institute of Modern Biological Techniques (RIMBT), University of Zanjan Zanjan 45371-38791 Iran; c Department of Chemistry Education, Farhangian University P. O. Box 14665-889 Tehran Iran

## Abstract

Expression of concern for ‘Recyclable mesalamine-functionalized magnetic nanoparticles (mesalamine/GPTMS@SiO_2_@Fe_3_O_4_) for tandem Knoevenagel–Michael cyclocondensation: grinding technique for the synthesis of biologically active 2-amino-4*H*-benzo[*b*]pyran derivatives’ by Mahdiyeh Partovi *et al.*, *RSC Adv.*, 2023, **13**, 33566–33587, https://doi.org/10.1039/D3RA06560J.

The Royal Society of Chemistry is publishing this expression of concern in order to alert readers that concerns have been raised regarding the reliability of the XRD data in Fig. 2.

The Royal Society of Chemistry has asked the affiliated institution to investigate this matter and establish whether the data provided by the authors provide an accurate representation of the experiments that were conducted and to confirm the integrity and reliability of the data provided.

An expression of concern will continue to be associated with the article until we receive conclusive evidence regarding the reliability of the reported data.

Laura Fisher

28th February 2025

Executive Editor, *RSC Advances*

